# Clinical Relevance of Mesenchymal- and Stem-Associated Phenotypes in Circulating Tumor Cells Isolated from Lung Cancer Patients

**DOI:** 10.3390/cancers13092158

**Published:** 2021-04-29

**Authors:** Evangelia Pantazaka, Vasileios Vardas, Argyro Roumeliotou, Stavros Kakavogiannis, Galatea Kallergi

**Affiliations:** Division of Genetics, Cell and Developmental Biology, Department of Biology, University of Patras, 26504 Patras, Greece; evapantazaka@upatras.gr (E.P.); up1088956@upatras.gr (V.V.); argyroumi@gmail.com (A.R.); up1052358@upnet.gr (S.K.)

**Keywords:** circulating tumor cells (CTCs), lung cancer, epithelial-to-mesenchymal transition (EMT), cancer stem cell (CSC), non-small-cell lung cancer (NSCLC), small-cell lung cancer (SCLC)

## Abstract

**Simple Summary:**

Lung cancer is the most frequent malignancy in the world. Most lung cancer patients are diagnosed at an advanced stage. To make matters worse, the survival of patients is very poor. Circulating tumor cells (CTCs), albeit rare, have been portrayed as essential players in the progression of lung cancer. It is definitely not easy being a CTC. First, they escape from the primary tumor, then they travel in the bloodstream, have to survive really harsh conditions, and finally, they form metastases. The adoption of epithelial-to-mesenchymal transition as well as cancer stem cell features has been suggested to allow CTCs to survive and metastasize. This review will focus on how these features can be used to estimate the prognosis of lung cancer patients.

**Abstract:**

Lung cancer is the leading cause of cancer-related mortality globally. Among the types of lung cancer, non-small-cell lung cancer (NSCLC) is more common, while small-cell lung cancer (SCLC) is less frequent yet more aggressive. Circulating tumor cells (CTCs), albeit rare, have been portrayed as essential players in the progression of lung cancer. CTCs are considered to adopt an epithelial-to-mesenchymal transition (EMT) phenotype and characteristics of cancer stem cells (CSCs). This EMT (or partial) phenotype affords these cells the ability to escape from the primary tumor, travel into the bloodstream, and survive extremely adverse conditions, before colonizing distant foci. Acquisition of CSC features, such as self-renewal, differentiation, and migratory potential, further reflect CTCs’ invasive potential. CSCs have been identified in lung cancer, and expression of EMT markers has previously been correlated with poor clinical outcomes. Thus far, a vast majority of studies have concentrated on CTC detection and enumeration as a prognostic tools of patients’ survival or for monitoring treatment efficacy. In this review, we highlight EMT and CSC markers in CTCs and focus on the clinical significance of these phenotypes in the progression of both non-small- and small-cell lung cancer.

## 1. General Background

Lung cancer is the type of cancer with the highest number of reported deaths worldwide. It is classified into two distinct subtypes based on cell size: non-small-cell lung cancer (NSCLC), including squamous cell carcinomas (SCC) and adenocarcinomas (ADC), which is responsible for the vast majority of cases, and small-cell lung cancer (SCLC) [1]. Collection of intact tumor samples is decisive for correct prognosis but is also an extremely difficult task, especially in advanced NSCLC [2]. SCLC is neuroendocrine and the most aggressive subtype with rapid development, early relapse after initial response to chemotherapy, fast and high metastatic potential, and increased mortality [3].

Circulating tumor cells (CTCs) are used for prognosis assessment, disease monitoring, and therapy management [4,5]. CTCs are released from the primary tumor or metastases and travel into patients’ bloodstream. In order to do so, they need to undergo phenotypic changes [6]. CTCs, as single cells or clusters, alter their genetic and phenotypic traits by expressing appropriate proteins in order to create beneficial niches, gain drug resistance, evade the immune system, and maximize their invasive potential for surviving in the hostile microenvironment of the blood [7]. These proteins can act as biomarkers for the detection, enumeration, characterization, and classification of CTCs into various subtypes.

These changes take place by a process, known as epithelial-to-mesenchymal transition (EMT). Under normal circumstances, EMT is essential for embryonic development and wound healing, whereas under pathological conditions it participates in tumor progression and dissemination of the malignant tumor cells [8]. EMT allows the survival of tumor cells; they avoid apoptosis as well as acquire chemoresistance [9]. During this process, cells lose their epithelial (E) properties and acquire a mesenchymal (M) phenotype with an increased invasive character [6]. More specifically, there is downregulation of E markers such as EpCAM and E-cadherin (E-cad) [10]. Additional loss of E markers includes proteins such as claudins and occludins, α and β-catenin, and cytokeratins (CK) [11]. On the other hand, upregulation of M markers such as N-cadherin (N-cad), fibronectin, matrix metalloproteinases, integrins α_v_ and β_1_, and smooth muscle actin takes place [9]. Transcriptional factors, such as Twist and Snail, also play a significant role in the survival and stability of CTCs in the bloodstream during EMT [12]. The expression levels of the cytoplasmic protein vimentin (Vim) are increased in tumor cells during EMT, suggesting a correlation with tumor progression. Elevated expression levels of thyroid transcription factor-1 (TTF-1), which participates in early human differentiation and morphogenesis of the developing lung and thyroid gland, has been indicated in multiple histologic subtypes of lung carcinomas [13]. Moreover, epidermal growth factor receptor (EGFR) also constitutes an essential factor in normal epithelial development as well as in the proliferation, motility, survival, and metastasis of tumor cells [14], and its expression has been associated with NSCLC aggressiveness [15]. Another protein whose levels have been shown to be increased during EMT is delta-like protein 3 (DLL-3), an inhibitory ligand of the Notch pathway receptor [16].

To maximize their aggressive capability, CTCs prefer to maintain a hybrid E/M population rather than changing to a complete M phenotype, and as such they can preserve their cell junction properties and, therefore, form clusters, while simultaneously gaining the necessary motility [17,18].

CTCs that survive in the blood circulation, finally, migrate to distant organs [19]. In fact, only a low number of CTCs with stem-cell-like characteristics are present in the blood stream [20]. It has been shown that in an adjuvant setting most of the CTCs are apoptotic [21]. Cancer stem cells (CSCs) are capable of surviving migration and, hence, constitute the main reason for cancer relapse and distant metastasis [22]. It has been suggested that CTCs with a stem phenotype are a result of CSCs’ transformation into CTCs, which initially stay in a state of dormancy and later become EMT-positive CSCs and initiate cancer relapse [23,24]. CTC clusters composed of heterogeneous cell populations, namely circulating tumor microemboli (CTM), can also be present in the bloodstream. It has been indicated that there is significant correlation between the presence of CTCs and CTM with M properties, and chemoresistance and poor prognosis [25,26,27,28,29].

Many different markers are involved in the formation of a stem phenotype in CTCs. Transcription factors NRF2 and OCT4 play a significant role in sustaining the self-renewing and chemoresistant properties of CTCs [6,30]. Furthermore, the cell surface molecule CD133 is a well-accepted marker [31]. CD133 plays a significant role in signaling pathways that are involved in CSC proliferation, such as the Wnt/β-catenin and PI3K-Akt signaling pathway or upregulation of FLICE-like inhibitory protein (FLIP) to inhibit apoptosis [32]. Additionally, *Bmi1* might function as a metastasis initiation gene by promoting EMT and stemness phenotype [33]. Similarly, aldehyde dehydrogenase (ALDH1) and CD44, are stem cell markers identified both in isolated CTCs and in the CTC-TJH-01 cell line, with an intermediate E/M phenotype, providing immune-escape characteristics [20,34]. Upregulation of ALDH1 has been correlated with the cancer cells’ acquired drug resistance [35], while CD44v isoforms play a significant role in stem phenotype by regulating self-renewal, tumor initiation, and metastasis [36].

**Figure 1:**
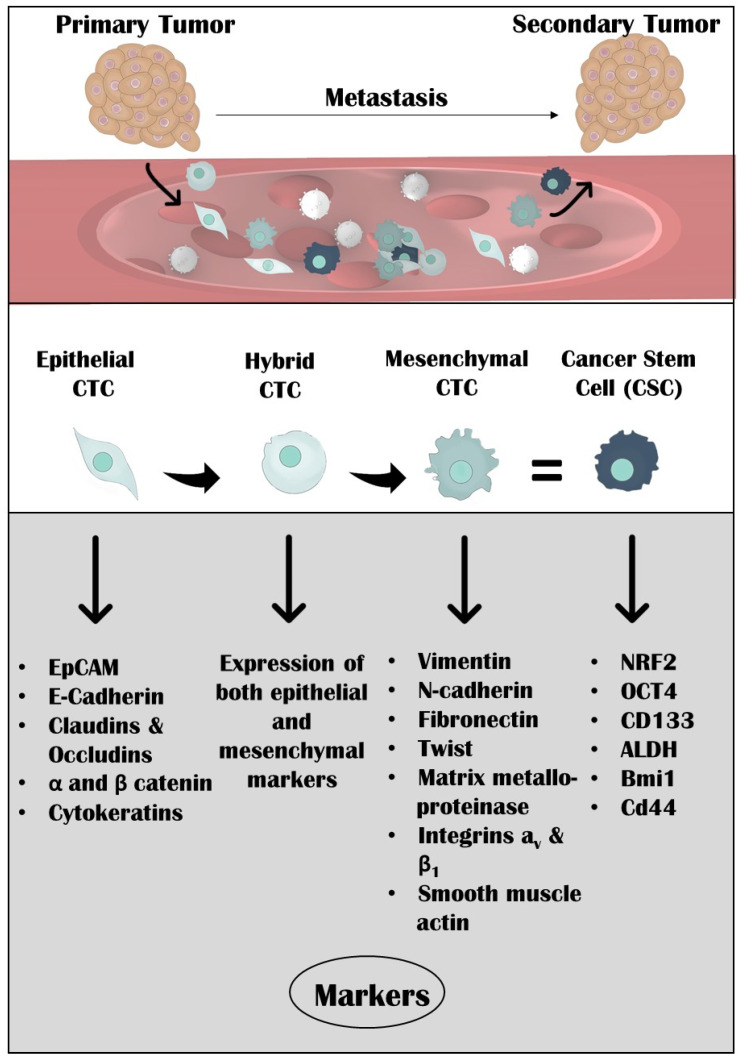
Mesenchymal and stem features in lung cancer patients.

In this review, we summarize the significance of the EMT and stem features of CTCs isolated from lung cancer patients (Figure 1) and focus on the clinical relevance of these phenotypes for future precision medicine.

## 2. Clinical Relevance of the Presence of CTCs in Lung Cancer

The increased number of CTCs has been shown to be associated with reduced patient survival and is, therefore, indicative of poor prognosis in both NSCLC and SCLC [37,38,39,40].

A number of studies in lung cancer have reported correlation between CTC counts and patients’ clinical outcome. In the study by Mayo et al. (2013), CTCs could be detected in the vast majority of later-stage patients at baseline (range 1–6 CTCs); CTCs were detected in 71% of surgically resected and 50% of unresectable stage IV patients (three CTCs on average for both cases). Overall, 86% and 83% of patients were positive for CTCs at baseline or post-surgery (without adjuvant therapy), respectively. Interestingly, the percentage of the detected CTCs decreased to 57% in patients upon therapy and reduced to 13% in those patients who responded to chemotherapy or tyrosine kinase inhibitors [41].

On the contrary, in the study by Que et al. (2019), which included 89 NSCLC patients, EpCAM^+^/EGFR^+^/CK^+^/CD45^−^ CTC counts were shown to be statistically lower in late-stage patients (mean number 14.6) compared to that in early stage ones (mean number 49.5) [34].

Reduced counts were seen following gemcitabine treatment. Thirty-nine NSCLC patients were included in the study by Liao et al. (2014). EpCAM^+^ CTCs were detected in 86% of patients (median 65 CTCs/mL, range 18–690), and when CTCs were evaluated at three different time intervals, they decreased as time progressed and were significantly lower in the gemcitabine-treated group compared to that in the non-treated, presumably due to inversion of HGF/cMET-induced EMT [42]. CTCs expressed lower *CK8, CK18*, and *CK19* genes in the gemcitabine group. EpCAM^+^ CTCs were also positively correlated with TNM stage in the gemcitabine-treated group. High CTC counts (>151) were identified as a prognostic factor (at follow-up) and associated with decreased median survival, which was slightly enhanced after gemcitabine treatment [42].

Another recent study by Chemi et al. (2019), reported that pulmonary-venous-derived CTCs (PV-CTCs) were detected in 48% (of 100 NSCLC patients), and this was an independent predictor of relapse in multivariate analysis [43].

A number of meta-analyses have suggested that having high CTCs prior to treatment is a prognostic factor of survival [44] and have identified the use of circulating tumor DNA (ctDNA) and CTCs as biomarkers for the detection of mutation status in NSCLC [45,46,47], while presence of CTCs also seems to indicate poor prognosis in SCLC [48].

## 3. Phenotypical Heterogeneity in CTCs from Lung Cancer

Soon it was very obvious in the field that a simple enumeration of CTCs was not enough, and attention was focused on the observed heterogeneity of CTCs. Representative studies are discussed herein.

In terms of EMT markers, Vim is among the most frequently examined. In the recent study by Zeinali et al. (2020), CTCs (PanCK^+^/DAPI^+^/CD45^−^) were detected in all 25 patients (average of 417 CTC/mL), while clusters of ≥2 CTCs were observed in almost all, i.e., 96% [49]. EpCAM^+^, Vim^+^, and EpCAM^+^/Vim^+^ subpopulations were also detected. A bit less than half of the isolated CTCs and clusters were Vim^+^ [49]. Higher numbers of clusters compared to single CTCs were associated with decreased progression-free survival (PFS), albeit not in a statistically significant manner [49]. Interestingly, the presence of Vim in CTC clusters implies that EMT phenotype in CTCs derived from lung cancer patients is strongly related to their metastatic potential.

In terms of CSC markers, *CD133* was evaluated in 45 patients (resectable or undergoing resection) in the study by Pirozzi et al. (2013). CK^+^ cells were detected in 24% of patients (range 2–10 cells). *CD133* was also expressed, but no statistically significant correlation was noted between the presence of CTCs and *CD133* expression [50].

Other studies have shown simultaneous expression of CSC and EMT characteristics in patients’ CTCs; however, this fact is not always related to the patients’ outcome. Specifically, CTCs were isolated from 13 metastatic NSCLC patients in the study by Koren et al. (2016) [33]. Analysis of gene expression in patients’ CTCs revealed that all patients expressed *EpCAM* and *ALDH1A1*, while *CD133* was detected in 50%, *Bmi1* in 80%, and *Twist1* in 40% of patients, confirming the presence of both EMT and CSC markers in NSCLC. Another recent study in 13 evaluable patient samples under anti-PD-1 nivolumab treatment, revealed that CTCs co-expressing PD-L1 with EMT markers such as N-cad or Vim ranged between 50% and 78% [51].

In the following sections, various markers and CTCs’ phenotypes are discussed, seeking to clarify their clinical significance as novel prognostic tools in patients with lung cancer.

## 4. Clinical Relevance of EMT and CSC Phenotypes in NSCLC Patients

A plethora of studies have identified correlations between EMT and/or CSC markers in CTCs and clinicopathological parameters of NSCLC patients (Table 1).

To begin with, a lot of interest has been shown toward elucidating EMT markers. Many studies have shown that the EMT phenotype can be related to distinct clinical characteristics of the tumor, such as proliferation status, clinical stage, etc. In a study by Peng et al. (2020) [53], CTCs from 84 patients were analyzed. The average positive rate of CTCs was 96% (median 5; range 0–68). EMT^+^ CTCs were detected in 73% of patients [53]. Increased EMT^+^ CTCs significantly correlated with lymphatic metastasis, tumor stage, and Ki67 overexpression. Patients with EMT^+^ CTCs were reported to have significantly shorter recurrence-free survival (RFS) and overall survival (OS). Univariate analysis not only identified a statistically significant association between the presence of EMT^+^ CTCs and both RFS (log-rank *p* < 0.001, HR = 2.743, 95% CI = 1.612–4.665) and OS (log-rank *p* = 0.007, HR = 2.236, 95% CI = 1.246–4.014) but also identified associations between RFS and OS and numerous other factors such as lymphatic metastasis, tumor size, smoking, tumor stage, degree of tumor differentiation, and Ki67 expression. In fact, patients with EMT^+^ CTCs in conjunction with high Ki67 expression levels in tumor tissues were reported to have worse RFS and OS. Based on multivariate analysis, however, only EMT^+^ CTCs were identified as an independent risk factor for RFS (log-rank *p* < 0.001, HR = 2.696, 95% CI = 1.554–4.677) and OS (log-rank *p* = 0.032, HR = 1.940, 95% CI = 1.060–3.550) [53].

The study by Zhang et al. (2019) is in line with the previous findings regarding the prognostic significance of EMT^+^ CTCs for NSCLC patients and especially in regard to metastasis. Eighty-five patients and twenty-five subjects with benign diseases were recruited in this study. Total CTCs (≥1 cell/5 mL blood) were detected in 86% of patients (median 5; range 0–57), with the CTC-positive rate in non-distant and distant metastases being 82% (median 4; range 0–17) and 90% (median 7; range 0–57), respectively. The median numbers of E^+^, E^+^/M^+^, and M^+^ CTCs were 2 (range 0–12), 2 (range 0–45), and 0 (0–10), respectively; the latter two being significantly higher compared to the values in benign patients [57]. Moreover, 32% of patients had CTCs of all phenotypes, 38% had two, and 17% had one phenotype, with the remaining patients being CTC negative. Interestingly, based on receiver operating characteristic curve analyses, E^+^/M^+^ CTCs were identified as predictors in distinguishing NSCLC from benign tumors, while M^+^ CTCs were identified as predictors of those with distant metastasis from those with non-distant metastasis [57]. Similarly, in another study 37 patients were recruited, and CTCs were detected in 89% of them. Univariate analysis showed that M^+^ CTCs were found mostly in patients with distant metastatic disease again compared to non-distant (*p* = 0.044) [6].

Vim, the popular EMT marker, is in some cases related to poorer clinical outcome, whereas in other studies expression of Vim is not a significant prognostic factor. In that case, it can be related to specific tumor cell subtypes, implying a correlation between EMT and genetic rearrangements during cancer evolution. In the study by Lindsay et al. (2017), 125 patients with treatment-naïve advanced disease were included. At baseline, CTCs were detected in 41% of patients (range 0–78 cells) and Vim^+^ CTCs were detected in 51% of the CTC-positive patients or 21% of all examined patients (range 0–35 cells) [37]. Treatment did not alter the percentage of patients with Vim^+^ CTCs. The presence of ≥5 total CTCs, detected in 19% of patients at baseline, conferred poor median PFS (*p* = 0.026, HR = 0.59, 95% CI = 0.37–0.94) and OS (*p* = 0.002, HR = 0.45, 95% CI = 0.28–0.75) based on univariate analysis. The presence of ≥5 CTCs was also identified by multivariate analysis as an independent prognostic factor for OS (*p* = 0.022, HR = 0.55, 95% CI = 0.33–0.92) but not for PFS (*p* = 0.118, HR = 0.68, 95% CI = 0.42–1.1). The presence of Vim^+^ CTCs was not correlated with poor prognosis, suggesting that the EMT phenotype did not offer any additional prognostic significance. When genetic subtypes were investigated, there was no change in Vim^+^ cells, but a statistically significant reduction in mean total CTCs in the *ALK*-rearranged subgroup. In the *KRAS*-mutated ADC, there was a statistically significant total lack of Vim^+^ cells. Interestingly, in the *EGFR*-mutated subgroup, a statistically significant increase of patients with total and Vim^+^ CTCs was observed, suggesting that this subgroup features EMT characteristics [37].

Another study where prognosis was shown to be subtype specific included 114 patients in the analysis. CTCs were present in 96% of the patients with 51% having ≥15/mL. In terms of phenotypes, 48% (of patients with ≥1 CTC) were M^+^ and 52% were E^+^ and Ε^+^/Μ^+^. Furthermore, 50% of patients were also PD-L1^+^ [58]. Total CTC and M^+^ counts were significantly decreased in the *EGFR*-mutant subgroup, compared to patients with the wild-type gene. M^+^ counts were statistically increased in the *BRAF*-mutant subgroup, while total CTC, M^+^, and PD-L1^+^ counts were statistically increased in the *KRAS*-mutant subgroup; in both cases compared to patients with the respective wild-type genes [58]. Patients with ≥15 CTCs had significantly shorter median disease-free survival (DFS) and OS. Patients with M^+^ and PD-L1^+^ CTCs also had significantly shorter median DFS. Multivariate analysis demonstrated that M^+^ CTCs (*p* = 0.003, HR = 0.330, 95% CI = 0.158–0.687), total PV-CTC counts (*p* = 0.005, HR = 0.274, 95% CI = 0.112–0.671), and disease stage (*p* = 0.013, HR = 0.344, 95% CI = 0.148–0.800) were independent factors of DFS, while disease stage was an independent prognostic factor of OS (*p* = 0.019, HR = 0.019, 95% CI = 0.046–0.762) [58].

The prognostic value of EMT phenotype and correlation with gene expression profile has also been demonstrated in the following study by de Miguel-Pérez et al. (2019). Ninety-seven patients with resectable tumors were enrolled. Detection of CTCs rather than specific phenotypic features was more informative in terms of prognosis assessment [15]. CTC values before surgical resection and during one and six months of follow-up were not statistically different between ADC and SCC. Interestingly, all patients with detected EMT^+^ (Vim^+^) CTCs also had EGFR^+^ CTCs. In ADC, the presence of CTCs one month after surgery was significantly associated with higher disease stages, and the presence of EMT^+^ CTCs before surgery, as well as six months after surgery was significantly associated with increased N stage and, thus, malignant progression. No such associations were observed in SCC patients [15]. EMT^+^ CTCs before surgery were related to gene expression of *AXL, IL6R*, and *GAPDH*, inversely correlated with *miR-155* expression, in ADC, whereas no such correlation was seen in patients with SCC. Presence of CTCs one month after surgery and high tissue expression of *AXL* were associated with shorter relapse-free survival (RFS) in ADC. In fact, in the multivariate analysis, the presence of CTCs one month after surgery was identified as an independent prognostic factor for RFS (*p* = 0.034, HR = 2.51, 95% CI = 1.07–5.87) [15]. The presence of CTCs six months after surgery (*p* = 0.017, HR = 10.8, 95% CI = 1.54–76.4) and tissue *AXL* gene expression (*p* = 0.017, HR = 15.7, 95% CI = 1.63–150.7) were associated with worse OS in ADC and were identified as independent prognostic factors based on multivariate analysis. Tumor size and N status were the two independent prognostic factors in terms of RFS in SCC patients, whereas no correlation between CTCs and RFS or CTCs and OS was observed [15].

The impact of EMT phenotype in CTCs was also confirmed in the study by Bian et al. (2020) [55]. Thirty-four patients were included in this analysis and CTCs were detected in 91% of patients (median 7, range 0–21 cells/7.5 mL) [55]. There were 53% CK^+^ CTCs and 91% CK^−^ (which represented EMT-derived CTCs). Briefly, ≥7 cells/7.5 mL of blood were detected in 56% of patients. A higher number of CTCs and higher counts of the subpopulation of CK^−^ CTCs were both statistically correlated with advanced tumor stages and the appearance of distant metastasis. Based on univariate analysis, a number of prognostic factors of PFS and OS were identified; the presence of ≥7 CTCs (OS; *p* = 0.003, HR = 2.554, 95% CI = 1.203–5.425 and PFS; *p* = 0.001, HR = 2.725, 95% CI = 1.273–5.831), ≥6 CK^−^ CTCs (OS; *p* = 0.004, HR = 3.455, 95% CI = 1.485–8.038 and PFS; *p* < 0.001, HR = 2.867, 95% CI = 1.329–6.185), distant metastasis, and therapy. Based on multivariate analysis, ≥6 CK^−^ CTCs and therapy were identified as independent prognostic factors for OS (CK^−^: *p* = 0.043, HR = 2.676, 95% CI = 1.034–6.927) and PFS (CK^−^: *p* = 0.044, HR = 2.849, 95% CI = 1.028–7.899) [55].

The presence of EMT^+^ CTCs and responsiveness to treatment is another open issue. Some studies attempted to address this question; however, data are limited, and there is an urgent need for further examination. Analysis of blood samples from 123 patients demonstrated the presence of CTCs in 69% of patients, and CTC count did not correlate with disease stage. Μ^+^ (E-cad^−^/Vim^+^) CTCs were detected in 46%, E^+^ (EpCAM^+^/Vim^−^) in 39%, and E^+^/Μ^+^ in 16% [56]. At baseline, patients with Vim^+^ CTCs had significantly poorer response to chemotherapy, and the presence of total CTCs and Vim^+^ CTCs was correlated with significantly shorter PFS (*p* = 0.040) compared to EpCAM^+^. Interestingly, the association observed for total CTCs was not detected after one cycle of chemotherapy.

Ten metastatic patients were included in a later study and were monitored before chemotherapy treatment and at two post-treatment time points. At baseline, CTCs were detected in 30% of patients (two patients had M^+^ and one had E^+^/M^+^ profile) and significantly increased to 88% of patients after chemotherapy (62% had an M^+^ or E^+^/M^+^ profile) [54]. The presence of CTCs at baseline was shown to be associated with faster progression. After chemotherapy treatment, the presence of EMT^+^ CTCs was identified as an unfavorable prognostic “trend”, associated with faster progression, poorer response, and shorter, yet not statistically significant, OS [54].

Finally, studies have demonstrated that EMT increases PD-L1 expression, ultimately leading to immune tolerance. Thirty treatment-naïve patients who had underwent curative surgical resection were included in the study by Manjunath et al. (2019) [10]. CK^+^/EpCAM^+^/CD45^−^ CTCs were detected in all patients with a mean count of 22 (median 19; range 12–45). Vim^+^ was detected in 97% of patients (median 23; range 0–61) and N-cad^+^ in 93% (median 20; range 0–63). PD-L1^+^ CTCs were found in all patients (median 36; range 8–89) and at a significantly higher rate than Vim^+^ and N-cad^+^ CTCs. PD-L1^+^/EMT^+^ (expressing both Vim and N-cad) CTCs were found in 87% of patients [10]. Noticeably, expression of PD-L1, Vim, and N-cad was significantly increased in CTCs compared to that in patient-matched tissues. Both total CTC and PD-L1^+^/EMT^+^ CTC counts were statistically significantly enhanced at higher stages of disease. Furthermore, the presence of ≥3 PD-L1^+^/EMT^+^ CTCs was associated (albeit not significantly) with more events of recurrence, and most importantly significantly shorter OS as determined by Kaplan–Meier analysis (log-rank *p* = 0.0368), after curative surgical resection [10].

In a recent study by Ntzifa et al. (2021), samples from 30 patients (at baseline, post first cycle with osimertinib treatment and at progression of disease, PD) were acquired and gene expression of the E markers (*CK8*, *CK18*, *CK19*), M/EMT markers (*Vim*, *Twist1*, *AXL*), and of the CSC marker *ALDH-1* were analyzed. Interestingly, only gene expression of *PD-L1* was significantly different between baseline and PD [59]. In addition, correlations between genes were observed at the different time points. In addition, E markers and *Vim* were co-expressed in 25% of the total cases studied; co-expression was observed in 20% of cases at baseline and in 27% at PD. CK^+^ (CK8/CK18/CK19) CTCs were detected in 76% of the available samples. High Vim^+^ CTC counts suggested a role for EMT during osimertinib treatment [59].

In terms of CSC markers, CD133 was evaluated in 43 patients. A variety of associations were observed. CD133^+^ CTCs were correlated with N-cad^+^ CTCs, while E^+^ CTCs were associated with treatment response. A significant difference was seen in respect to CK^+^, N-cad^+^, and CD133^+^ and later stage of disease [52]. CD133/CK^+^ ratio and M^+^ presence were associated with shorter PFS based on Kaplan–Meier analysis (*p* = 0.003, HR = 4.43 and *p* = 0.03, HR = 2.63, respectively) [52].

Overall, it is evident that there is a lot of heterogeneity between the reported studies on NSCLC, even concerning the chosen CTC detection method (Table 1). The number of patients in half of the studies was relatively high, although bigger cohorts can be further designed. In terms of the evaluated markers, Vim has been mostly studied and correlated with survival. Even so, it is still far from providing data with prognostic significance values probably due to the variety of identification assays employed in these studies. Interestingly, attention is also gradually being paid on CSC markers, as well as other potentially interesting molecules, such as PD-L1, bringing players of the immune system into the game.

## 5. Clinical Relevance of EMT and CSC Phenotypes in SCLC Patients

Interestingly, to date, there has been limited attention toward investigating links between CTCs’ phenotype and clinical outcomes of the disease in SCLC (Table 2).

One of the earliest studies proposed that EMT occurs differently in the population of CTCs, whereas CTM, which lack apoptotic cells, show enhanced survival that might contribute to metastasis [63]. The following study from the same research team, revealed that CTC count was an independent variable, associated with significantly decreased PFS (*p* = 0.011, HR = 2.01, 95% CI = 1.17–3.46 at baseline) and OS (*p* = 0.002, HR = 2.45, 95% CI = 1.39–4.30 at baseline and *p* = 0.03, HR = 4.1, 95% CI = 1.1–15.1 after one cycle of chemotherapy) [64]. Moreover, CTM and apoptotic (assigned by fragmented and condensed nuclear morphology) CTCs were associated with poor OS (*p* = 0.006, HR = 2.25, 95% CI = 1.26–4.21 and *p* = 0.001, HR = 2.66, 95% CI: 1.49–4.74, respectively) before chemotherapy. They were characterized as independent prognostic factors [64]. Nevertheless, there was no examination between different CTCs subpopulations, based on the expression of Ki67 (proliferative), Bcl-2 (highly expressed in SCLC), and Mcl-1 (non-apoptotic) markers, and clinical values such as PFS or OS [64].

SCLC tumors have been shown to express neuroendocrine peptides [65]. This study by Messaritakis et al. (2017) investigated TTF-1^+^/CD45^−^, CD56^+^/CD45^−^, and TTF-1^+^/CD56^+^ phenotypes in CTCs. The study suggested that TTF-1^+^/EpCAM^−^ CTCs probably represent CTCs undergoing EMT [66]. An increased number of CTCs at baseline was defined as an independent factor, correlating to decreased PFS (*p* = 0.048, HR = 1.9, 95% CI = 0.9–3.9), while increased number of CTCs at PD was also an independent factor associated with lower OS (*p* = 0.041, HR = 2.1, 95% CI = 0.9–5.3). No significant association between CTC subpopulations and clinical values was reported [66].

In addition to the above reports, where CTC subpopulations did not reveal significant prognostic value, there are other studies showing that EMT phenotype could be important for clinical evaluation of the patients. The prognostic value of EMT phenotype in CTCs has been shown in a following study, in patients treated with front-line chemotherapy, subpopulations of CK^+^/Ki67^+^ and CK^+^/Vim^+^ CTCs were detected even in patients without any detectable CTCs by CellSearch (CK^+^/EpCAM^+^ or Vim^+^/EpCAM^+^) [13]. CTC counts with CellSearch emerged as an independent prognostic factor for reduced PFS at baseline and reduced OS at PD (*p* = 0.032, HR = 1.9, 95% CI = 0.7–3.6 and *p* = 0.043, HR = 2.1, 95% CI = 1.0–4.5, respectively), while only the increased number of Vim^+^ CTCs at baseline and of M30^−^ (non-apoptotic) CTCs at PD were identified as independent prognostic factors associated with decreased OS (*p* = 0.023, HR = 4.6, 95% CI = 1.2–16.8 and *p* = 0.009, HR = 6.4, 95% CI = 1.6–25.8, respectively) [13].

An ensuing study from the same research team, analyzed the effect of second-line therapy with an anti-angiogenic agent (pazopanib) in CTC subpopulations of patients with recurrent and resistant/refractory disease [60]. Only CK^+^/Vim^+^ CTCs after one treatment cycle and CTCs counts at PD were evaluated as independent factors linked to shorter OS (*p* < 0.001, HR = 7.9, 95% CI = 2.9–21.8 and *p* = 0.005, HR = 2.0, 95% CI = 1.0–6.0, respectively), whereas CTC counts at baseline were evaluated as an independent factor with shorter PFS (*p* < 0.001, HR = 4.9, 95% CI = 2.3–10.6) [60]. In addition, the two above studies [13,60] further support the variant heterogeneity of CTCs subpopulations as presented in Hou et al. (2012) [64].

Bcl-2 is highly expressed in SCLC patients. Results revealed a phenotypic heterogeneity of CTCs, presented as Bcl-2^+^/Vim^+^, Bcl-2^+^/Vim^−^, Bcl-2^+^/CK^+^, Bcl-2^+^/CK^−^, and Bcl-2^+^/M30^−^, before and after front-line treatment [62]. Bcl-2 could be detected in CTCs without any correlation with E (CK^+^/Bcl-2^+^/CD45^−^, CK^−^/Bcl-2^+^/CD45^−^) or M (Vim^+^/Bcl-2^+^/CD45^−^, Vim^+^/Bcl-2^−^/CD45^−^) markers, suggesting that this phenotypic heterogeneity could be associated with undergoing EMT [62]. A significant association between Bcl-2^+^/CD45^−^ cells at baseline with decreased PFS (*p* = 0.005, HR = 4.5, 95% CI = 1.6–12.9) and OS (*p* = 0.001, HR = 4.3, 95% CI = 1.2–7.0) was revealed, with Bcl-2^+^/CD45^−^ being identified as an independent factor for both PFS and OS. In addition, the presence of Bcl-2^+^/CD45^−^ CTCs after one chemotherapy cycle was related to low OS (*p* = 0.007, HR = 13.9, 95% CI = 2.1–33.2) and was again identified as an independent factor [62]. Changes of Bcl-2^+^/CD45^−^ CTCs before and during treatment have been suggested to be related to treatment efficacy [62].

Based on indications that the Notch pathway is related to EMT and stem [67] phenotype, expression of DLL3, alone and co-expressed with Vim in CTCs from patients receiving front-line chemotherapy, and its possible clinical relevance was examined in another study by Messaritakis et al. (2019) [16]. A plethora of heterogeneous phenotypes such as CK^+^/DLL3^+^, CK^+^/DLL3^−^, CK^+^/Vim^+^/DLL3^+^, CK^+^/Vim^−^/DLL3^+^, and CK^+^/Vim^+^/DLL3^−^ was revealed. Τhe subpopulation of DLL3^+^/CD45^−^ CTCs was found to be an independent prognostic factor. At baseline, it was also significantly linked to decreased PFS (*p* = 0.005, HR = 10.8, 95% CI = 2.1–56.4), while at PD it was associated with shorter OS (*p* = 0.016, HR = 28.2, 95% CI = 2.0–39.1) [16]. DLL3 silencing was shown to restrain proliferation, migration, and the EMT process in SCLC cell lines [68], providing a possible explanation that overexpression of DLL3 may result in poor clinical outcomes.

Finally, a more recent study revealed a correlation between Vim^+^ CTCs to liver metastases (*p* = 0.002). Vim^+^ CTCs in patients with advanced lung cancer at baseline were identified as independent factor of low PFS (*p* = 0.013, HR = 2.756, 95% CI = 1.239–6.131) and subsequently of poor prognosis [61].

In terms of CSC phenotypes, an interesting study examining CSC markers (SOX2 and CD44) and EMT (E-cad, EpCAM, CK 8/18/19, Vim and c-MET) in tumors from SCLC patients’ biopsies found no association with CTC counts at baseline or with OS [69]. In contrast, expression of both high levels of c-MET and low levels of E-cad in patient tumors (c-MET^High^/E-cad^Low^) was associated with better OS (*p* = 0.007, HR = 0.30, 95% CI = 0.13–0.72) and lower number of baseline CTCs (*p* = 0.09), following Cox regression analysis and two-sided Fishers exact test, respectively [69].

Overall, despite the fact that SCLC patients are the population with the greater clinical need compared to NSCLC due to the aggressiveness of the disease, fewer studies have been conducted so far concerning CTCs’ identification and characterization (Table 2). Interestingly, limited number of labs are focused on evaluating treatment efficacy (Table 2). Despite the decreased number of studies, all have identified factors of prognostic significance and Vim was portrayed in more than half of them, albeit at different treatment time points. Therefore, there is a lot of ground to be covered in SCLC, but this fact does not diminish the importance of the past efforts.

## 6. NSCLC vs. SCLC and CTCs

EMT and CSC characteristics are present in both NSCLC and SCLC patients’ CTCs. The prognostic value of these phenotypes has been evidenced in many studies. However, due to the variety of different evaluation approaches and identification methods, there are controversial results. Furthermore, the number of studies in SCLC is limited compared to that in NSCLC. Further studies especially in SCLC will clarify the potential clinical use of these biomarkers.

Interestingly, propagation of CTCs in vitro via the generation of stable CTC cell lines has been reported for a number of tumors including NSCLC and SCLC [34,70,71]. This is a useful tool for screening expression of potentially interesting biomarkers, pathways involved, and even potential drugs for the treatment of lung cancer.

Furthermore, propagation of CTCs in vivo by the generation of CTC-derived xenografts/explants is also reported [72,73]. These explants overcome the issue of tumor tissue availability encountered with patient-derived xenografts, which is crucial when it comes to advanced-stage SCLC patients compared to NSCLC patients. Explants can be established from CTCs collected at different time points during follow-up. As CTCs recapitulate the tumor’s heterogeneity, so do the expected explants. Of course, this can also pose a problem, as heterogeneity reduces reliability and any high-throughput chance these models might have, and ultimately translation to clinical usage.

Ex vivo expansion of CTCs from patients with SCLC has also been recently [74] reported. Initial findings have reported the existence of EMT and of other currently investigated markers such as PD-L1, but this will have to be further examined. Ex vivo manipulations and subsequent in vivo testing can also increase the options of the available toolkit, which can further facilitate evaluation of treatment regimens, chemoresistance, and the identification of other key players. A limitation regarding expansion of CTCs is the need for high numbers of tumor cells. Evidently, all the aforementioned tools offer great potential, provided, there is solid information that phenotypic plasticity is maintained, so it can mirror the effects of single or clustered CTCs.

## 7. Conclusions

Liquid biopsy can be used for tumor diagnosis, monitoring response to therapeutic regimens, and ultimately evaluating drug efficacy or development of chemoresistance [75]. It is rapid, non-invasive, and serially obtained as compared to tissue biopsies.

CTCs and ctDNA present in lung cancer patients’ blood could be used in concert as complementary approaches, and none has to win over the other [76]. In NSCLC, ctDNA could be used to track and examine the effect of “druggable” mutations, e.g., of EGFR, and together with CTCs to identify expression of potentially crucial players and of changes in tumor characteristics before, during, and after appropriate treatments. In SCLC, liquid biopsy in the form of CTCs and ctDNA could be used for deciding on a treatment regimen, prognosis, and treatment efficacy.

In order to find a needle (CTC) in the haystack (billions of normal hematopoietic cells), a very good detection system is required. Detection of CTCs in lung cancer is already challenging; CTCs usually lack epithelial characteristics and, thus, identification of CK^+^ CTCs can be difficult. A number of detection methods are currently being used and more are being developed. Although evaluation of the existing detection platforms is beyond the scope of this review, it is important to stress once more that the only FDA-approved method of CTC isolation/enrichment has a major disadvantage, i.e., failure of capturing a subset of CTCs devoid of an epithelial phenotype due to EMT. As this is a drawback of label-dependent technologies, further advances are urgently needed, or alternatives need to be sought.

Relevant to lung cancer is also the fact that only a small number of CTCs are detected at early stage. This problem can be overcome by identifying other/more specific markers, as discussed in the review. When identification of subpopulations of CTCs, representative of tumor behavior, is correlated with predictive and/or prognostic value, as reviewed herein, then it could be of particular clinical significance.

In truth, liquid biopsy needs a bit more time to prove its full potential outside the clinical trial settings, and it seems that all the right actions are in motion. The high heterogeneity of CTCs observed in many types of cancer, including NSCLC and SCLC, can often be linked to their metastatic potential, allowing real-time monitoring of the tumor. Studies investigating heterogeneity both in CTCs and tumors can help toward recognizing all important players and consequentially developing novel diagnostics and specialized treatment. It is evident more than ever that simple CTCs’ enumeration, no matter how informative, is not enough. However, enumeration of CTCs and correlation with survival has put liquid biopsy on the map as a potentially powerful way of allowing the genetic and molecular characterization of tumors.

Indeed, clinical significance of CTCs is not yet fully exploited, but further understanding of the importance of certain processes including EMT and metastasis, dissection of CTC biology, and identification of new important players is bound to be translated to clinical studies. It remains to be seen when and how it will be employed in, hopefully, near-future clinical care. The goal of personalized oncology is to match a lung cancer patient, notwithstanding the tumor’s heterogeneity, with suitable biomarkers and subsequently treatment regimen.

## Figures and Tables

**Table 1:** Epithelial-to-Mesenchymal Transition (EMT) and Cancer Stem Cells (CSC) biomarkers expressed in Circulating Tumor Cells (CTCs) and associated with clinical values of Non-Small-Cell Lung Cancer (NSCLC) patients.

Detection Method	Biomarker(s)				Patients, *n*	Stage of Disease/Stage of Treatment	Clinical Significance	Prognostic Significance	Refs
	CTC	EMT	CSC	OTHER					
Ficoll-Hypaque	PanCK EpCAM	N-cad	CD133		43	IIB–IV	CD133/CK^+^: ↓ PFS M^+^: ↓ PFS		[52]
CellSearch	≥5	Vim			125	IIIB–IV Baseline	≥5 CTC: ↓ PFS, ↓ OS	≥5 CTCs (OS)	[37]
CellSieve™ microfilters	CK 8/18/19 EpCAM CD45 (-)	Vim N-cad		PD-L1	30	I–IIIA	≥3 PD-L1^+^/EMT^+^: ↓ OS		[10]
Carcinoma cell enrichment and detection kit with MACS Technology	CK7/8/18/19	Vim		EGFR	97	I–III ADC	CTCs: ↓ RFS, ↓ OS	CTCs (1 month post-surgery; RFS and 6 months post-surgery; OS), *AXL* expression (OS)	[15]
CanPatrol^TM^	CK19	Twist		Ki67	84	I–IV	EMT^+^: ↓ RFS, ↓ OS	EMT^+^ (RFS, OS)	[53]
Immunomagnetic negative depletion	CEA CK19	Vim Snail1-2 ZEB1-2 Twist1-2			10	Baseline Post-surgery	EMT^+^: ↓ OS (not statistically significant)		[54]
SE-i·FISH^®^CTC kit	CK18	CK^−^			34	IIB–IVB	CTCs: ↓ PFS, ↓ OS EMT^+^: ↓ PFS, ↓ OS	Univariate (OS, PFS): ≥7 CTCs, ≥6 EMT^+^ CTCs, distant metastasis, therapy Multivariate (OS, PFS): ≥6 EMT^+^ CTCs, therapy	[55]
TelomeScan F35	CK19/ PanCK EpCAM E-cad	Vim			123	I–IV	CTCs: ↓ PFS EMT^+^: ↓ PFS		[56]
CanPatrol^TM^	CK8/18/19 EpCAM	Vim Twist1			85	I–III	E^+^/M^+^: distinguish malignant vs. benign M^+^: distinguish distant vs. non-distant metastasis		[57]
CanPatrol^TM^	CK8/18/19 EpCAM	Vim Twist1			37	I–IV	M^+^: distinguish distant metastasis		[6]
CanPatrol^TM^	CK8/18/19 EpCAM	Vim Twist		PD-L1	114	I–III	CTCs: ↓ DFS, ↓ OS M^+^: ↓ DFS PD-L1: ↓ DFS	≥15 PV-CTCs (DFS), M^+^ (DFS), disease stage (DFS, OS)	[58]
Parsortix system ISET	CK8/18/19	Vim Twist1 AXL	ALDH1	PD-L1 PIM-1	25–30	Baseline Post 1st cycle PD	Vim^+^ and osimertinib treatment		[59]

Abbreviations: DFS; disease-free survival, OS, overall survival; PFS, progression-free survival; RFS; recurrence-/relapse-free survival.

**Table 2:** Epithelial-to-Mesenchymal Transition (EMT) and EMT-related biomarkers expressed in Circulating Tumor Cells (CTCs) and associated with clinical values of Small-Cell Lung Cancer (SCLC) patients.

Detection Method	Biomarker(s)	Stage of Disease/Stage of Treatment	Patients, *n*	Clinical Significance		Prognostic Significance	Refs
				PFS	OS		
Ficoll-Hypaque	non-apoptotic (M30^−^)	LD-SCLC and ED-SCLC, PD	108	-	↓	M30^−^	[13]
Ficoll-Hypaque, anti-CD45 magnetic beads	Vim	LD-SCLC and ED-SCLC, baseline	108	-	↓	Vim^+^	[13]
		Post 1st treatment cycle	Baseline (56), after one cycle (35), PD (45)	-	↓	Vim^+^	[60]
		Stage IIIA/IIIB and Stage IV baseline	61 (44 SCLC and 17 ADC)	↓	-	Vim^+^	[61]
Ficoll-Hypaque	Bcl-2	LD-SCLC and ED-SCLC baseline	Baseline (66), after one chemotherapy cycle (59), PD (38)	↓	↓	Bcl-2^+^	[62]
		LD-SCLC and ED-SCLC Post 1st treatment cycle	Baseline (66), after one chemotherapy cycle (59), PD (38)	-	↓	Bcl-2^+^	
Ficoll-Hypaque	DLL3	LD-SCLC and ED-SCLC baseline	Baseline (108), after one chemotherapy cycle (68) PD (48)	↓	-	DLL3^+^	[16]
		LD-SCLC and ED-SCLC PD	Baseline (108), after one chemotherapy cycle (68) PD (48)	-	↓	DLL3^+^	

Abbreviations: ED, extensive disease; LD, limited disease; OS, overall survival; PD, disease progression; PFS, progression-free survival.
